# Intersession Consistency of Single-Trial Classification of the Prefrontal Response to Mental Arithmetic and the No-Control State by NIRS

**DOI:** 10.1371/journal.pone.0037791

**Published:** 2012-07-23

**Authors:** Sarah D. Power, Azadeh Kushki, Tom Chau

**Affiliations:** 1 Insitute of Biomaterials and Biomedical Engineering, University of Toronto, Toronto, Ontario, Canada; 2 Bloorview Research Institute, Holland Bloorview Kids Rehabilitation Hospital, Toronto, Ontario, Canada; Institution of Automation, CAS, China

## Abstract

Near-infrared spectroscopy (NIRS) has been recently investigated for use in noninvasive brain-computer interface (BCI) technologies. Previous studies have demonstrated the ability to classify patterns of neural activation associated with different mental tasks (e.g., mental arithmetic) using NIRS signals. Though these studies represent an important step towards the realization of an NIRS-BCI, there is a paucity of literature regarding the consistency of these responses, and the ability to classify them on a single-trial basis, over multiple sessions. This is important when moving out of an experimental context toward a practical system, where performance must be maintained over longer periods. When considering response consistency across sessions, two questions arise: 1) can the hemodynamic response to the activation task be distinguished from a baseline (or other task) condition, consistently across sessions, and if so, 2) are the spatiotemporal characteristics of the response which best distinguish it from the baseline (or other task) condition consistent across sessions. The answers will have implications for the viability of an NIRS-BCI system, and the design strategies (especially in terms of classifier training protocols) adopted. In this study, we investigated the consistency of classification of a mental arithmetic task and a no-control condition over five experimental sessions. Mixed model linear regression on intrasession classification accuracies indicate that the task and baseline states remain differentiable across multiple sessions, with no significant decrease in accuracy (p = 0.67). Intersession analysis, however, revealed inconsistencies in spatiotemporal response characteristics. Based on these results, we investigated several different practical classifier training protocols, including scenarios in which the training and test data come from 1) different sessions, 2) the same session, and 3) a combination of both. Results indicate that when selecting optimal classifier training protocols for NIRS-BCI, a compromise between accuracy and convenience (e.g., in terms of duration/frequency of training data collection) must be considered.

## Introduction

Many individuals with severe and multiple motor disabilities rely entirely on access devices (e.g., mechanical switches, eye-trackers) for communication and environmental control. However, for individuals who have retained no reliable, voluntary motor control in any part of the body (e.g., as in total locked-in syndrome), conventional access devices, which are primarily movement-based, are ineffective. Brain-computer interface (BCI) technologies are controlled via brain activity alone, and may provide these individuals with an alternative, movement-free means of access. Researchers have recently begun investigating the potential of near infrared spectroscopy (NIRS), an optical imaging technology that can be used to assess functional activity in the cerebral cortex via measurement of the hemodynamic response (see [1] for description of fundamental principles), for use in a safe, noninvasive BCI system.

Generally, a user controls a BCI output by consciously eliciting distinct, reproducible patterns of activation in a particular region of the brain, which is usually achieved by performing different mental tasks (e.g., motor imagery). The system then detects and interprets these task-induced patterns of activation, and produces the appropriate command signal to control a connected external device (e.g., computer cursor) in the way the user intended. Each distinct, intentionally-generated pattern the system is able to recognize can represent a different user command (e.g., left-hand motor imagery  =  “cursor left”, right-hand motor imagery  =  “cursor right”).

Thus, essential to NIRS-BCI development is the ability to accurately detect and classify patterns of activation associated with different mental tasks. The prefrontal cortex is an attractive measurement region due to the absence of hair, which can significantly degrade NIRS signals [2]. Previous studies have demonstrated the ability to classify prefrontal cortical responses to several different mental tasks (e.g., mental singing [3]–[5], affective picture viewing [6], verbal tasks [7]), with the most prevalent, and promising, being mental arithmetic (MA) [3]–[5], [7]–[9]. However, there is a paucity of literature regarding the consistency of these responses, and the ability to classify them on a single-trial basis, over multiple sessions. This is important when moving out of an experimental context and toward a practical system, where performance will have to be maintained over long periods of time. In all prefrontal NIRS-BCI studies to date, all data used for single-trial classification have either been collected in a single session [5], [6], [10] or have been collected over multiple sessions but pooled into a single data set for analysis [3], [4]. There have been no multi-session results reported for mental arithmetic, or any other prefrontal task.

**Figure 1:**
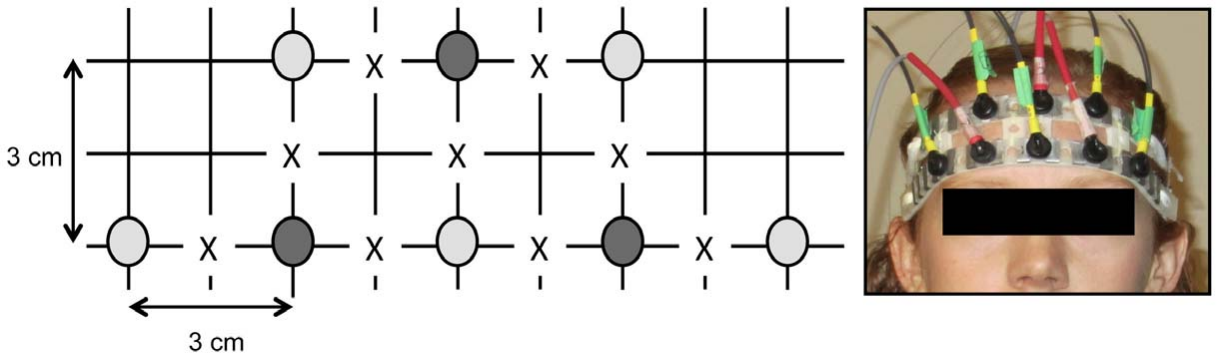
Source-detector configuration. The dark shaded circles represent photomultiplier tube detectors, while the light shaded circles represent NIR source pairs (containing one 690 nm and one 830 nm source). X’s represent points of interrogation.

When considering the consistency of responses across sessions, there are two main questions of interest. The first is whether or not the hemodynamic response to the activation task can be distinguished from a “baseline” condition consistently across sessions. Even though previous research has demonstrated that the response to a particular task can be detected in a single session, it is possible that participants could habituate to the task over time, resulting in a degradation of classification accuracy and system performance across many sessions. If this is the case, then such a task would clearly be unsuitable for use in a practical BCI system. If the response to the activation task does indeed occur consistently over sessions, however, the second question of interest is whether or not the measured response looks the same each time; i.e., are the spatiotemporal characteristics of the response which best distinguish it from the “baseline condition”, consistent from session to session. The consistency of the measured response may be affected by intersession variability in user-related (e.g., task strategy, fatigue, motivation, baseline characteristics), as well as environmental (e.g., distractions) and instrumentation-related (e.g., sensor coupling), factors. Such variability in response characteristics would have important implications for BCI system design, particularly in the adopted classification strategies, and especially in terms of classifier training protocols (e.g., will a classifier trained using “old” data remain effective across sessions or does it need to be re-trained before each use?).

**Figure 2:**
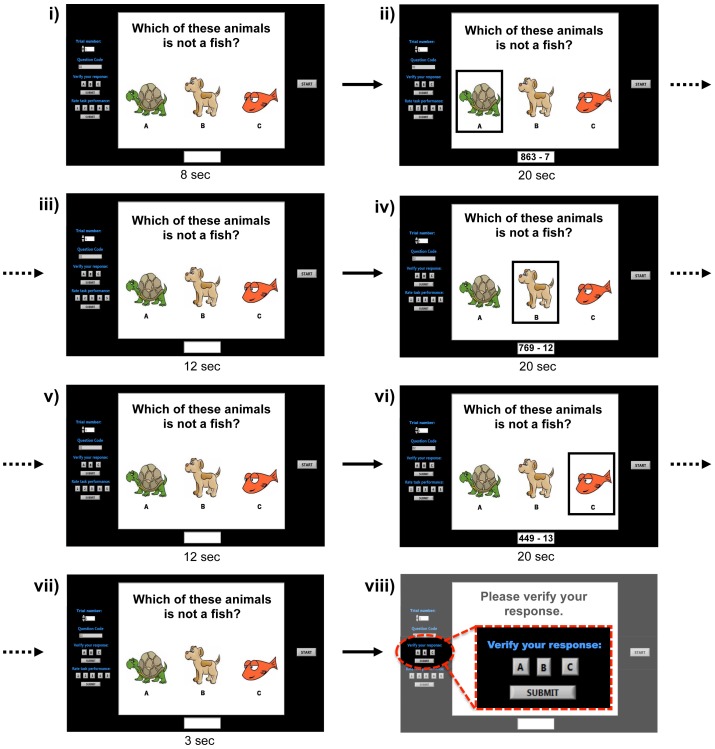
Example trial cues and timing diagram. In this example, the user would perform mental arithmetic during intervals ii) and iv) to select options A and B, and would remain in the “no-control” state during all other intervals. In viii), the participant would verify his/her response via the controls shown.

In light of the above, we investigated the consistency with which a mental arithmetic task could be differentiated from a “no-control” baseline condition over five experimental sessions. The term “no-control” refers to the natural state existing when the user is not consciously modulating their brain activity for the purpose of controlling the BCI output (e.g., during periods of thinking, monitoring, composing or daydreaming) [11]–[13]. In particular we address the following questions:

1. Does the ability to distinguish mental arithmetic and a “no-control” baseline condition using signals acquired from the prefrontal cortex via NIRS remain consistent across multiple sessions?
2. If the answer to #1 above is affirmative, do the response characteristics that allow best discrimination of the mental arithmetic task from a “no-control” baseline condition remain consistent across multiple sessions?

**Figure 3:**
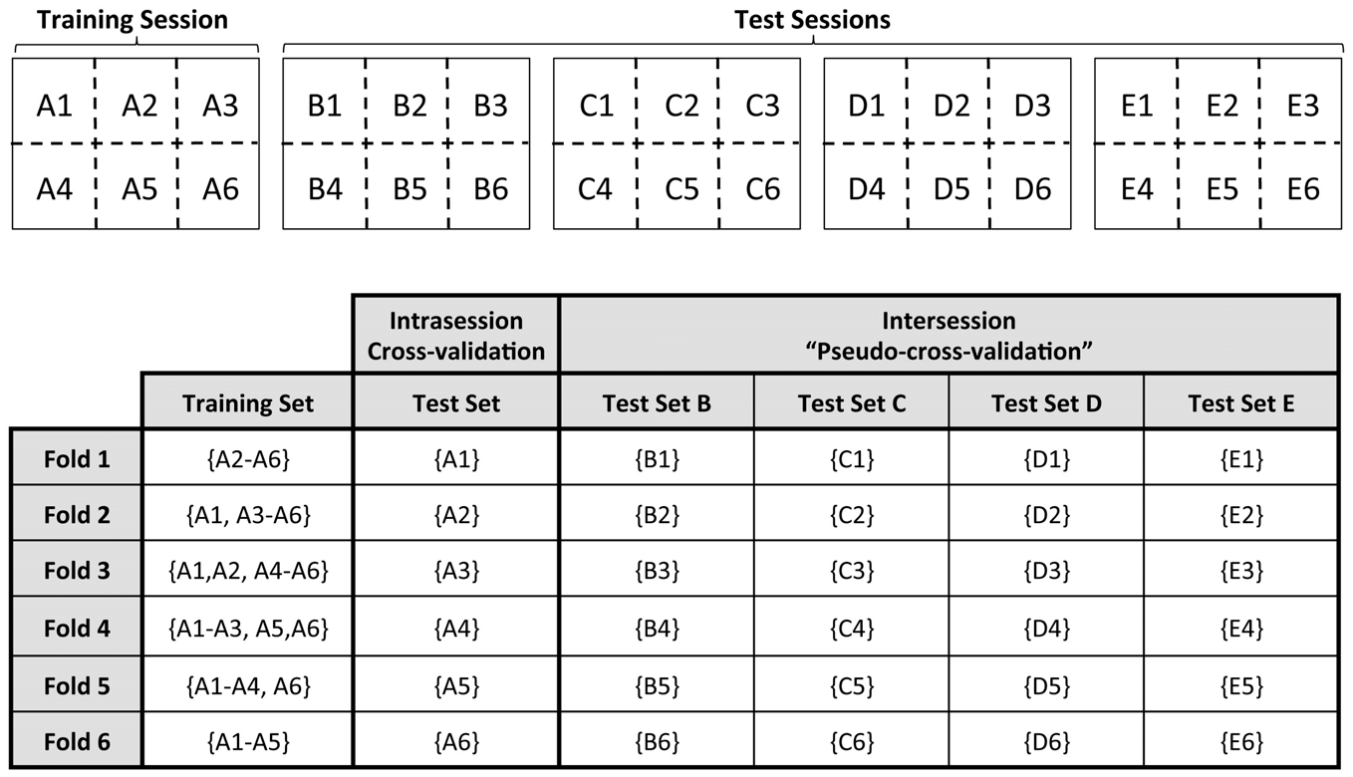
A single run of 6-fold intrasession cross-validation and intersession “pseudo-cross-validation”. In this example, Session A is the training session, and the remaining four sessions are for testing only. Training and test session data are all divided randomly into six sets. For each of the six folds, a classifier is trained on five sets of the training session data (a different set is left out at each fold). For the intrasession cross-validation, the resulting classifier is then tested on the remaining set from the training session; for the intersession “pseudo-cross-validation”, the classifier is tested on a single set from each of the four test sessions (a different set at each fold).

Furthermore, based on the answers to these questions we investigated the effectiveness of a number of different classifier training protocols which differed in terms of the training sets used. Otherwise the classification procedure remained the same in terms of feature selection and learning algorithm. The training variations included scenarios in which 1) training and test data came from different sessions, 2) training and test data came from the same session, and 3) a combination of 1) and 2).

**Table 1:** Classifier Training Conditions.

Condition	Training Set Composition	Size of training set (*n_total_*)
I	All samples obtained from a single, dedicated training session, recorded on a different day thanthe current test session	96
II	The first *n_curr_* samples from the current test session	*n_curr_*
III	The first 96 −*n_curr_* samples from a single, dedicated training session - recorded on a different day thanthe current test session - combined with the first *n_curr_* samples from the current test session	96
IV	All samples obtained from the combination of two separate, dedicated training sessions,recorded on different days (than both one another and the current test session)	2 × 96 = 192

*n_total_* denotes the overall size of the training set.

## Materials and Methods

### Ethics Statement

Approval for this study was obtained from the Research Ethics Board of both Holland Bloorview Kids Rehabilitation Hospital and the University of Toronto. Participation was voluntary and all participants provided informed, written consent.

**Table 2:** Within- and Across-Session Classification Accuracies By Participant.

	Classification Accuracy (%)					
Participant	Session 1	Session 2	Session 3	Session 4	Session 5	LS Mean (95% CI)
1	83.1	76.8	76.5	82.2	91.2	82.0 (77.2, 86.7)
2	77.4	79.2	68.8	73.1	67	73.1 (68.4, 77.8)
3	63.8	66.4	–	71	69.1	67.6 (62.3, 72.8)
4	65.3	64.0	65.7	64.0	52.9	62.4 (57.7, 67.1)
5	72.0	69.5	–	60.5	66.7	67.2 (61.9, 72.5)
6	77.1	79.4	71.5	79.2	80.3	77.5 (72.8, 82.2)
7	59.1	64.3	67.4	73.3	65.1	65.8 (61.1, 70.5)
8	73.9	74.8	75.0	80.8	81.8	77.2 (72.5, 81.9)
9	72.3	90.1	79.0	79.8	71.8	78.6 (73.9, 83.3)
**LS Mean**	**71.6**	**73.8**	**70.6**	**73.7**	**71.8**	**72.6 (70.1, 75.1)**

Individual session accuracies obtained by averaging over 25 runs of 6-fold cross-validation (training and test sets taken from the same session).

### Participants

Ten able-bodied adults (mean age = 23.2
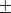
4.5 years; two male) were recruited from the students and staff at Holland Bloorview Kids Rehabilitation Hospital (Toronto, Canada). Individuals were excluded from participation if they had any condition that could adversely affect either the measurements or their ability to follow the experimental protocol (specifically, any metabolic, cardiovascular, respiratory, psychiatric, or drug- or alcohol-related conditions). All participants had normal, or corrected-to-normal, vision. Participants were asked to refrain from consuming caffeinated or alcoholic beverages, or smoking cigarettes, for at least 3 hours prior to the experimental sessions.

**Figure 4:**
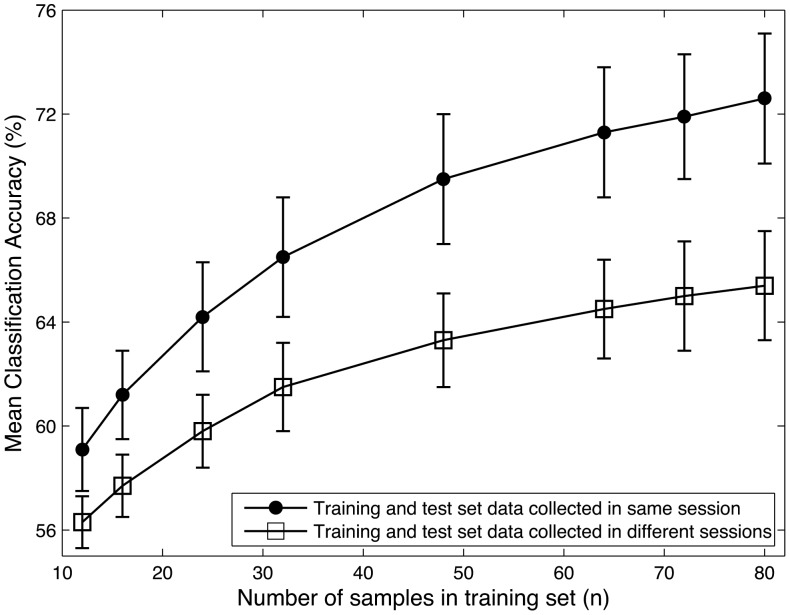
Across-participant least squares mean classification accuracy for the intra- and intersession cross-validation procedures, plotted against training sample size, n. Results shown are for the case of 6-fold cross validation, which corresponds to a training sample size of 80. Bars indicate 95% confidence intervals.

### Instrumentation

Signals were acquired using a dual-wavelength multichannel frequency-domain NIRS instrument (Imagent Functional Brain Imaging System, ISS Inc., Champaign, IL). A specially made flexible headband was used to secure ten NIR sources and three photomultiplier tube detectors against the participant’s forehead, as shown in Figure 1. So that each location could be probed by both wavelengths simultaneously, the ten sources were grouped into five pairs, each containing one 690 nm and one 830 nm source. The headband was positioned on the participant’s forehead such that the centre row of optodes was in line with the bridge of the participant’s nose, and the bottom row sat just above the eyebrows. Nine distinct locations within a 27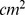 trapezoidal area were probed, as shown in Figure 1. In terms of optode placement, though there is currently no standardized placement scheme for NIRS measurements [14], it is generally accepted that 3 cm is the ideal source-detector separation for measuring cortical hemodynamics [15]. Therefore, in the given configuration we considered only signals arising from source-detector pairs (henceforth referred to as “channels”) with a separation of 3 cm, which yielded a total of 18 channels (i.e., 3 detectors×3 locations per detector×2 wavelengths per location). Each channel was sampled at 31.25 Hz.

### Mental Arithmetic Task

For the mental arithmetic task, participants performed a sequence of simple mathematical calculations, which began with the subtraction of a small number (between four and thirteen) from a three digit number, and continued throughout the task interval with successive subtractions of the small number from the result of the previous subtraction; that is, the difference of one subtraction became the minuend for the next, with the subtrahend remaining the same (e.g., 753−13 = 740, 740−13 = 727, 727−13 = 714, etc.). The initial subtraction for a given response period was displayed visually on the screen.

### Experimental Protocol

Each participant completed five experimental sessions which were conducted on different days over, at most, a month. During each session, participants performed a total of 32 trials. In each trial, participants sat in front of a computer and were visually and aurally presented with a question and three possible responses. After the question had been displayed on the screen for 8 seconds, the three choices were highlighted in sequence for periods of 20 seconds each, separated by 12 second intervals in which none of the three responses were highlighted (to allow prefrontal hemodynamics to return to a baseline state). The timing of an example trial is shown in Figure 2. A unique question was used for each of the 32 trials of a single session, however the same 32 questions were used in each of the five sessions. The periods in which one of the three responses were highlighted represent the “system-vigilant” periods in a BCI system [3].

Participants were instructed to answer the questions by performing the MA task throughout the intervals in which their desired responses were highlighted. There was not necessarily a single correct answer for a given question; there could be one, two, three or no correct answers. During the intervals in which they did not wish to make a selection, participants were not required to control their mental activity in any particular way, but rather were told to allow natural thought patterns to occur without restriction. This represented the natural baseline state that we refer to as the “no-control (NC)” state. To ensure that the data during the system-vigilant periods could be properly labeled as MA or NC, we included only questions with obvious answers (see example in Figure 2), and participants were asked to explicitly verify their selection(s) at the end of each trial.

The 32 trials, each with three system-vigilant periods, yielded a total of 96 20-second samples per session, which were divided evenly between MA and NC. Thus, for each session, 48 samples each of MA and NC were obtained. Each session was approximately 1.5 hours in duration.

**Figure 5:**
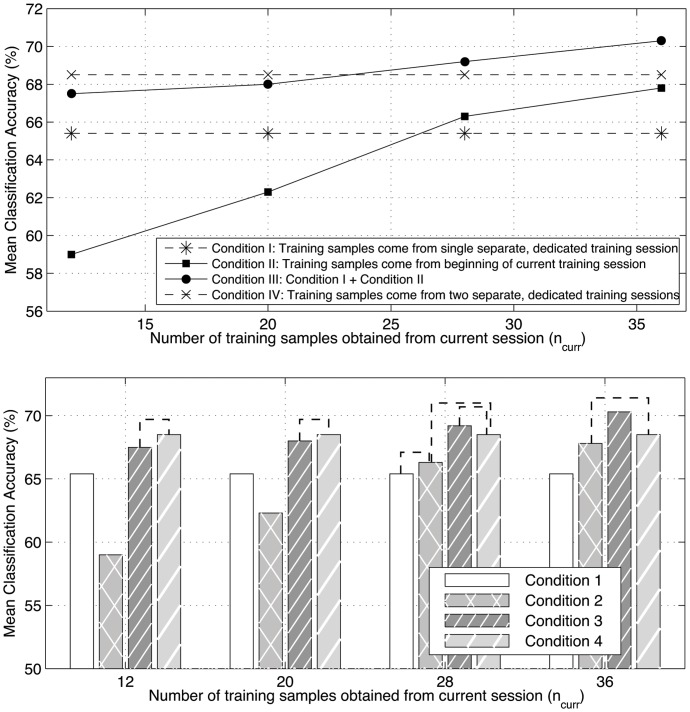
Across-participant least squares mean classification accuracy for the four classifier training protocols under consideration for different values of 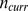. All pairwise differences between conditions are statistically significant except those bars joined by dashed lines in the bottom figure (5% significance level).

Note that a unique initial subtraction was displayed for each response period of a given session (regardless of whether the participant was “supposed” to select the response or not) for a total of 96 unique initial subtractions per session. The same 96 initial subtractions were used in each of the 5 sessions.

### NIRS Data Pre-processing

For each 20-second sample of MA or NC arising from the system-vigilant periods, each of the 18 signals (i.e., 2 wavelengths at 9 interrogation locations) was first normalized by the mean and standard deviation of the 90-second period preceding the end of the interval. The samples were then linearly detrended over the same 90-second period. These steps were taken in order to account for any subtle inter-trial differences in sensor coupling or placement, or instrumentation-related drift. The 20 second response intervals were then extracted and given the appropriate label of MA or NC in preparation for classification.

The raw normalised light intensity signals for each response interval were low-pass filtered in order to mitigate physiological noise due primarily to respiration (0.2–0.3 Hz) [16], cardiac activity (0.8–1.2 Hz) and the Mayer wave (approximately 0.1 Hz) [17]. A 3rd-order Chebyshev type II filter was designed with cut-off frequency at 0.1 Hz, stop frequency at 0.5 Hz, pass-band loss of no more than 6 dB, and at least 50 dB of attenuation in the stop-band [3]. Relative changes in the concentrations of oxy- and deoxy-hemoglobin (HbO_2_ and HHb, respectively) were then calculated for each measurement location using the dual wavelength light intensity signals and the modified Beer-Lambert law [18], as shown in (1), (2) and (3):
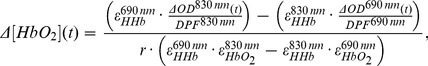
(1)

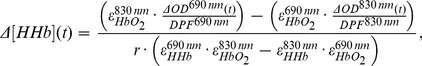
(2)where
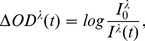
(3)and


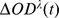
  =  change in optical density of light at wavelength 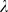,


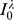
  =  mean light intensity at wavelength 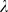,


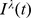
  =  light intensity at wavelength 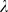,


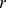
  =  source-detector separation distance (3 cm in this case),


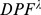
  =  differential pathlength factor in the human adult head at wavelength 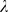,


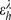
  =  extinction coefficient of chromophore 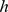 (i.e., HbO_2_ or HHb) at wavelength 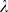.

Values for the extinction coefficients (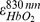 = 1.0507, 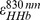 = 0.7804, 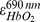 = 0.3123, 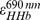 = 2.1382) and differential pathlength factors (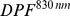 = 5.86, 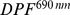 = 6.51) were taken from the literature [19], [20]. 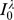 was calculated over the 8 second period preceding the 20 second response interval.

### Feature Extraction

The hemodynamic response to mental tasks typically appears as an increase in 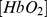, with a corresponding decrease in 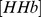, peaking at approximately 5–8 s after onset of activity [21]. Note, however, that other trends have been reported [9], [22]. To capture this behavior, we considered as features the signal slope over a number of possible time windows within the 20 second response interval [3]. Each time interval was defined by a start time and an end time. Start times ranged from 0 to 15 s in 5 second increments, while end times ranged from 5 to 20 s, also in 5 second increments. All possible combinations of start and end times, where the latter exceeded the former, were considered as valid time intervals for feature calculation. In total, ten different time windows were considered. Thus, the resultant feature pool consisted of 180 candidate features comprising the slope of the regression line fit to each of the 18 concentration signals over each of the 10 time windows. The different time windows were considered in order to capture the unique temporal response for each individual, as there could be inter-subject variability in the time required for the hemodynamic response to peak, and/or in the number of peaks.

**Figure 6:**
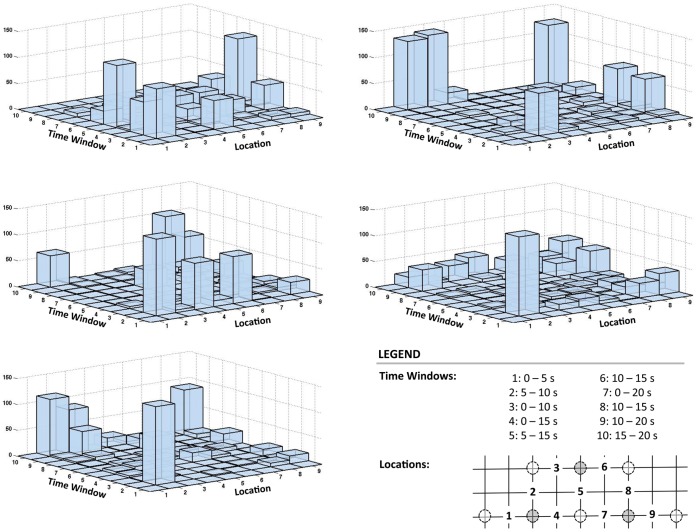
Intersession variability in spatiotemporal response characteristics. Histograms show frequency of feature selection during 25 runs of 6-fold cross-validation (feature subset dimensionality of 5) for each of the 5 sessions for Participant #1. Features (all representing slopes of regression lines fit to the concentration signals) are indicated by a combination of location and time window. Note that no distinction is made between 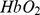 and HHb in the histogram, but for this participant, 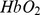 features were selected approximately two times as often as HHb features (on average across sessions). This is reflective of the overall trend; on average across participants and sessions, 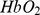 features were selected 1.75 times as often as HHb features.

### Feature Selection

Optimal feature subsets were selected using a sequential feedforward feature selection algorithm. To evaluate the fitness of a candidate feature subset, 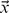, linear discriminant analysis (LDA) was performed on the training set to obtain the weight vector, 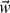, and threshold constant, 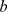, that provided optimal separation of the classes when projected into a one-dimensional feature space via.
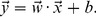
(4)


The Fisher criterion in this projected space, 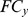, served as the fitness function and was computed via
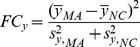
(5)where 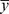 and 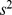 represent the mean and variance, respectively, of the indicated class. For a given run of the feature selection algorithm, the feature subset, 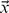, yielding the highest projected Fisher criterion, 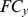, as estimated from a training data set, was selected as the optimal feature set and used in the classification of a test data set. The different classification problems explored are described in the next section.

### Classification

#### Consistency of response detection across sessions

To answer our first question regarding the consistency with which the MA and NC states can be distinguished across multiple sessions, we performed 25 runs of 6-fold cross-validation (using the feature selection method described above, and an LDA classifier) separately for each of the five experimental sessions, for each participant. Note that for each fold of the cross-validation, the test set was involved in neither the feature selection nor classifier training. Mixed model linear regression was used to determine if accuracies changed significantly across the five sessions.

#### Consistency of response characteristics across sessions

To answer our second question regarding the consistency of the discriminatory response characteristics across multiple sessions, we examined whether or not classification accuracy changed significantly when training and test data came from different sessions as compared to when they came from the same session. If the discriminatory response characteristics are consistent from session to session, then one would expect similar classification accuracies in both scenarios.

Thus, we repeated the cross-validation classification procedure described above, where, as usual, the training and test data came from the same session, and compared the classification results to those of a “pseudo-cross-validation” (also 25 runs) where the training and test sets were taken from different sessions. In the “pseudo-cross-validation” analysis, each of the five sessions acted separately as the training session, with each of the four remaining sessions being used for testing. This resulted in a total of 20 training/test session combinations per participant. More specifically, for a given training session, the resulting classifier from each fold of the pseudo-cross-validation was used to test a different set of test data from each of the four remaining sessions. This intersession “pseudo-cross-validation” procedure is clarified in Figure 3. To investigate the effect of the number of training samples, the k-fold cross-validation and “pseudo-cross-validation” analyses were repeated for different values of 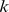. The values of 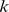 used resulted in training sample sizes of 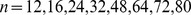.

Repeated measures mixed model linear regression was used to compare the classification results obtained when the training and test sets came from the same session (i.e., the intrasession cross-validation) to those obtained when the training and test sets came from different sessions (i.e., intersession “pseudo-cross-validation”), for each value of 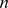.

**Figure 7:**
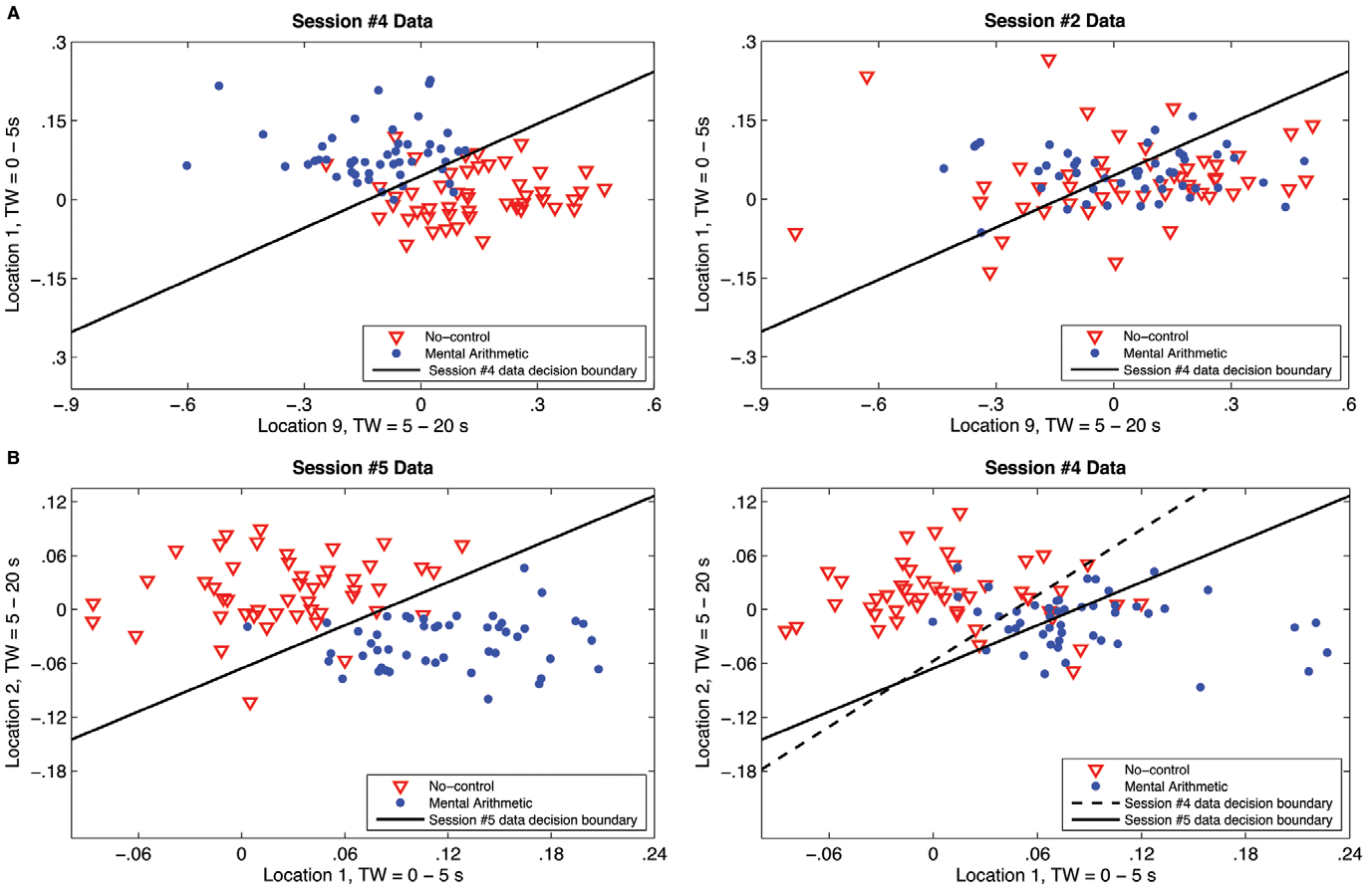
Example of intersession variability in response characteristics. Data shown are for Participant #1. TW  =  time window. A) The two juxtaposed graphs exemplify a case in which a feature space that allows good separation of the NC and MA states in one session is completely ineffective for separating the two classes in a different session. B) The two graphs portray a case in which a given feature space allows good separation of the NC and MA states in two separate sessions, however the specific distributions of each class are shifted between the two sessions such that different decision boundaries are needed to provide optimal classification accuracy. The dimensions of all axes are 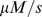 (i.e., change in concentration per time).

#### Different classifier training protocols

Finally, based on the results of the intra- and intersession cross-validation analysis, we investigated various training protocols suitable for implementation in a practical NIRS-BCI system that differed only in terms of the training sets. We were interested in scenarios in which the training and test data came from the same session, different sessions, and a combination of both. Specifically, we considered four different training conditions as outlined in Table 1.

For all four training protocols of interest, the feature selection algorithm described previously was performed on the training set under consideration, and the resulting optimal features of the training data were used to train the classifier. To reduce variability and obtain more generalizable results, the analysis was performed (on an individual participant basis) for every possible combination of training and test sessions for a given condition. That is, for Conditions I and III, each session served independently as the test session, with each of the four remaining sessions serving as separate, dedicated training sessions. For Condition IV, each session again served independently as the test session, with each pairwise combination of the four remaining sessions used for training. For Condition II, each session was tested only once, using a single classifier trained on data from that same session. Note that to keep the size of the test set constant across conditions, in all cases the test set comprised the last 60 samples from the test session under consideration. Repeated measures mixed model linear regression was used to compare the four conditions for different values of 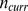.

The amount of training data used in each of the four conditions was limited to what would be practical for a real-life BCI system. In particular, for Conditions II and III, the number of training samples taken from the current test session (i.e., 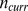) was limited to what could be collected in at most 30 minutes (including equipment set-up), which is comparable to the calibration time (not including equipment set-up) reported for some EEG-based online systems [23]. Values of 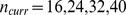 were considered.

For the conditions in which training data from one or more dedicated training sessions were used (i.e. Conditions I and IV), all 96 samples from the given session(s) were used for training. As mentioned, it took approximately 1.5 hours to collect 96 samples (including equipment set up and breaks). Based on informal feedback, this seemed to be approaching the limit of what participants could comfortably tolerate in a single session.

For Condition III, training samples consisted of 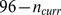 from a dedicated training session and 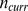 from the current test session, so that 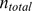 would be equal to that of Condition I. In this way, any change in accuracy between Conditions I and III could be attributed to the inclusion of current session training data rather than differences in training sample size.

## Results

Data for one participant who reported having considerable difficulty remaining attentive to the experimental protocol were excluded from analysis; results for the remaining nine participants are reported. P3 reported feeling ill during one of the five sessions (unrelated to the experimental protocol); the session was cut short and the data were excluded from analysis. A significant portion of Session #3 data for P7 was corrupted due to an instrumentation failure; the entire session was excluded from analysis.

The detailed intrasession cross-validation results for the case of 6-fold cross-validation (which corresponds to 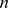 = 80 samples in the training set) are reported in Table 2. Overall LS mean classification accuracy (across participants and sessions) is 72.6%. Mixed model linear regression showed that there was no significant effect of session on classification accuracy (p = 0.67). Note that we also evaluated the classification ability of each of the two chromophores individually, by performing the 6-fold cross-validation with feature pools only comprising either the HbO_2_, or HHb, features. The two chromophores yielded similar classification accuracies on average (HbO_2_ = 61%, HHb: 62%), with neither performing as well individually as when the two were considered together.

Figure 4 shows the least squares mean (across participants and sessions) classification accuracies (error bars represent 95% confidence limits) obtained for the intra- and intersession k-fold (pseudo-) cross-validation analysis for different values of 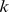. In the figure, mean classification accuracy (%) is plotted against the number of training samples in the training set (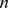), rather than against 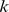. Mixed model repeated measures linear regression revealed that for all values of 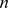 there was a significant effect of condition, i.e., accuracies obtained when the training and test sets came from the same session were significantly greater than those obtained when the training and test sets came from different sessions (p
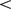
0.0001 for all 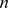).

Figure 5 shows the least square mean classification accuracies obtained for the four different classifier training schemes, plotted against 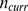. Note that accuracies for Conditions I and IV, in which no training samples are taken from the current test session, are independent of 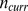. The bottom plot of Figure 5 indicates the statistical significance for pairwise comparisons between conditions for the different values of 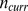.

Note that training on three (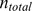 = 288; ls mean accuracy = 69.1%) and four (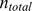 = 384; ls mean accuracy = 69.3%) separate, dedicated training sessions did not result in significantly higher accuracies (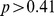) than training on two (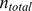 = 192; ls mean accuracy = 68.5%).

All results reported are for a prescribed feature subset dimensionality of 5.

## Discussion

### Consistency of Response Detection Across Sessions

This study represents the first investigation into the consistency of single-trial classification of task-induced prefrontal activity using NIRS over multiple sessions. The results of the intrasession cross-validation confirm that the response to the mental arithmetic task does indeed exist in the prefrontal cortex, and remains distinguishable from the baseline state using NIRS signals, across multiple sessions. The results of the repeated measures mixed model linear regression indicate that there is no significant deterioration of classification accuracy over sessions, suggesting that factors such as habituation due to task repetition do not adversely affect the prefrontal response to the MA task. While these results also suggest that there was no significant *increase* in task differentiability over multiple sessions, it is important to note that participants were not given any sort of feedback regarding their performance on a trial-to-trial basis, and therefore did not undergo training of any kind. There is no evidence that precludes participant improvement upon provision of formal feedback training.

### Consistency of Response Characteristics Across Sessions

Though the results of the intrasession cross-validation show that the mental arithmetic task can be consistently distinguished from the baseline state across multiple sessions, the results of the intersession “pseudo-cross-validation” suggest that the characteristics of the response change from session to session. For a given training sample size, 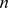, the classification accuracies achieved in the “pseudo-cross-validation”, when the training and test sets came from different sessions, were significantly lower than those achieved in the intrasession cross-validation, when the training and test sets came from the same session. Clearly, models trained using data taken from the same session as the test data generally characterized the response more accurately than models trained using data taken from a different session. This suggests that the discriminatory response characteristics varied from session to session.

These quantitative results are supported by further investigation of the spatiotemporal characteristics of the response. The histograms in Figure 6 show the distribution of features selected during the 25 runs of 6-fold intrasession cross-validation for each of the five sessions for Participant #1. Features are depicted as a combination of location of interrogation and time window (no distinction is made between HbO_2_ and HHb signals). Note the obvious contrast in the overall distribution of selected features across the five sessions. Though there are some consistencies across sessions in terms of individual features selected, there are also some clear differences; for example, 1) “Location 2, Time Window 2” was selected frequently in Sessions 1, 3, 4 and 5, but was not selected at all in Session 2, and 2) “Location 9, Time Window 10” was selected frequently in Sessions 2 and 5, somewhat frequently in Sessions 3 and 4, but not at all in Session 1. Further visualization of the intersession variability in response characteristics is depicted in Figure 7. Figure 7a shows an example in which a feature space that allows clear separation of the mental arithmetic and no-control classes in one session, is completely ineffective for separating the classes in a different session. Figure 7b gives an example in which a given feature space allows separability of the classes in two different sessions, but the specific distribution of the response in this feature space has shifted between the two sessions, and thus different decision boundaries are needed to optimize classification.

The observed intersession variation in the prefrontal response characteristics may be attributable to user-related (e.g., task strategy, fatigue [24], motivation, attention [25], baseline characteristics, emotional state [26]), environmental (e.g., auditory distractions [27]) and/or instrumentation-related (e.g., small variation in sensor placement, coupling) factors. Such factors have also been noted as sources of variance in EEG-BCI systems [28].

These intersession results have serious implications for the development of a practical NIRS-BCI system, especially in terms of the classifier training protocols adopted. To date, most studies investigating the feasibility of NIRS-BCI have been preliminary investigations of task differentiability in which classification is performed offline, usually using cross-validation with large training sets [4], [6], [10], [18]. Though this is a very important first step in BCI development, this method of analysis (i.e., a large amount of training data collected during the same session as the “test” data) does not reflect what would be possible in a practical situation - users cannot be expected to sit through extended calibration sessions each time they wish to use their device. As conjectured in [18], an obvious alternative is to collect the classifier training data during a separate training session, eliminating the need for lengthy calibration sessions before each subsequent use of the system. The results presented above, however, suggest that this method may result in significantly lower performance than that forecasted by offline cross-validation analysis. The intersession findings further show that as the size of the training set (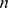) decreases, so does classification accuracy. This, of course, is not unexpected, but it implies that if the number of training samples is reduced to what could reasonably be collected during a pre-use calibration session, performance would again be lower than what conventional offline cross-validation methods using large data sets might suggest.

### Different Classifier Training Protocols

The classification results achieved for the four training conditions (see Table 1) correspond well to the results of the intra- and intersession cross-validation analyses discussed above. Classification accuracies obtained for Condition II, where the training data came from the same session as the test data, matched or even exceeded the accuracies of the two scenarios where the training and test data came from different sessions (Conditions I and IV), even though the size of the training set (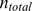) for Condition II was several times smaller. However, Figure 5 indicates that when training and test data derive from different sessions, intersession variation can be mitigated and comparable accuracies achieved (as in Condition IV) as long as a sufficiently large training set is available. Given that it would be much more convenient to do a one-time collection of a large amount of training data (even if it had to be spread over multiple sessions, as necessitated by the user’s specific needs and abilities) than collect even moderate amounts of training data before every use, the former may actually be the preferred compromise between accuracy and practical convenience. For example, even if 200 training samples are needed from previous training sessions to achieve the same accuracy that can be achieved with only 40 training samples collected at the beginning of the current session, it would be much more convenient to do this one time collection of 200 samples than to have to collect 40 samples before *each use of the system*.

Accuracy seems to be further improved by augmenting the data collected during a separate, dedicated training session with a small number of calibration samples from the current test session. Note that even though 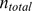 was equal for Conditions I and III, accuracies for the latter condition (which included samples from the current test session) significantly exceeded the former for all values of 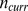 considered; for larger values of 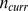, Condition III accuracies even met/exceeded those for Condition IV, despite 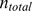 being smaller. However, as discussed, collecting these samples before every use would represent an inconvenience for the user. A compromise between accuracy and convenience - based on the user’s preferences, needs and abilities - would have to be made in terms of how much current session calibration data, if any, to collect.

### Study Limitations

One limitation of this study is the lack of immediate feedback to the user. Consequently, it is unclear how the findings translate to a practical scenario in which the user will receive feedback of the classification result after each response. Indeed, it has been suggested that EEG signal characteristics undergo a shift when moving from the non-feedback to feedback conditions [29]; no similar investigation has yet been done for NIRS signal characteristics. If such a shift does occur, it may be necessary to collect a set of training data (giving no feedback) to initially train the classifiers, then using the trained classifiers to provide feedback, collect another set of data to retrain the classifiers under the feedback condition. This latter set of training data would then more closely represent the practical-use scenario.

It is also worth noting that we cannot be completely certain that the measured responses used in the classification of mental arithmetic and no-control represent localized cortical activity, as it is possible that, for some participants and/or locations, the observed trends could be the product of a task-induced global systemic response that affects the entire brain. Of course, this would be a significant problem if our aim was to, for example, map cortical function or compare cortical activation among disease groups. However, given that our objective in BCI design is simply to differentiate between the two mental states, not to draw any conclusions about underlying neural mechanisms, the physiological origin of the detected hemodynamic response is inconsequential. Furthermore, it is impossible to know what effect task-induced frontalis muscle tension [30] might have had on the optical signals collected. Though we are confident that no related motion artefacts exist in the signals, there could theoretically be other effects related to potential spectroscopic changes due to tissue compression, blood flow, etc. While this tension, if present at all, would have been minimal and unlikely to have a significant influence, it cannot be conclusively ruled out. Though not usually considered, this is a concern for all NIRS studies of mental tasks based on prefrontal activity.
